# Targeting the insulin-like growth factor-1 receptor in MTAP-deficient renal cell carcinoma

**DOI:** 10.1038/s41392-019-0035-z

**Published:** 2019-01-25

**Authors:** Jihao Xu, Wen-Hsin Chang, Lon Wolf R. Fong, Robert H. Weiss, Sung-Liang Yu, Ching-Hsien Chen

**Affiliations:** 1 Division of Nephrology, Department of Internal Medicine, University of California Davis, Davis, CA USA; 2 Institute of Molecular Medicine, National Taiwan University College of Medicine, Taipei, Taiwan; 3 Department of Experimental Therapeutics, Division of Cancer Medicine, The University of Texas MD Anderson Cancer Center, Houston, TX USA; 4 Medical Service, Department of Veterans’ Affairs Northern California Health Care System Center, Sacramento, CA USA; 5 Comprehensive Cancer Center, University of California Davis, Sacramento, CA USA; 6 Department of Clinical Laboratory Sciences and Medical Biotechnology, National Taiwan University College of Medicine, Taipei, Taiwan

**Keywords:** Urological cancer, Molecular biology, Nephrology

## Abstract

Renal cell carcinoma (RCC) has emerged as a metabolic disease characterized by dysregulated expression of metabolic enzymes. Patients with metastatic RCC have an unusually poor prognosis and near-universal resistance to all current therapies. To improve RCC treatment and the survival rate of patients with RCC, there is an urgent need to reveal the mechanisms by which metabolic reprogramming regulates aberrant signaling and oncogenic progression. Through an integrated analysis of RCC metabolic pathways, we showed that methylthioadenosine phosphorylase (MTAP) and its substrate methylthioadenosine (MTA) are dysregulated in aggressive RCC. A decrease in MTAP expression was observed in RCC tissues and correlated with higher tumor grade and shorter overall survival. Genetic manipulation of MTAP demonstrated that MTAP expression inhibits the epithelial-mesenchymal transition, invasion and migration of RCC cells. Interestingly, we found a decrease in the protein methylation level with a concomitant increase in tyrosine phosphorylation after MTAP knockout. A phospho-kinase array screen identified the type 1 insulin-like growth factor-1 receptor (IGF1R) as the candidate with the highest upregulation in tyrosine phosphorylation in response to MTAP loss. We further demonstrated that IGF1R phosphorylation acts upstream of Src and STAT3 signaling in MTAP-knockout RCC cells. IGF1R suppression by a selective inhibitor of IGF1R, linsitinib, impaired the cell migration and invasion capability of MTAP-deleted cells. Surprisingly, an increase in linsitinib-mediated cytotoxicity occurred in RCC cells with MTAP deficiency. Our data suggest that IGF1R signaling is a driver pathway that contributes to the aggressive nature of MTAP-deleted RCC.

## Uncovering metabolic changes that underlie kidney cancer

A receptor that is triggered by an enzyme deficiency in kidney cancer could act as an anticancer drug target. Ching-Hsien Chen of the University of California Davis and colleagues in the USA and Taiwan found that renal cell carcinomas are deficient in the enzyme methylthioadenosine phosphorylase (MTAP). This deficiency, which correlates with higher tumour grade and shorter overall survival, leads to the activation of type 1 insulin-like growth factor-1 receptor (IGF1R). This in turn activates signaling pathways that support cancer cell survival, growth, and invasiveness. The team found that a selective IGF1R inhibitor, called linsitinib, suppressed colony-forming ability and reduced cell motility in renal carcinoma cells. The findings suggest that IGF1R signaling drives pathways that contribute to the aggressive nature of renal carcinoma cells lacking MTAP.

## Introduction

Kidney cancer (or renal cell carcinoma, RCC) is increasing in incidence, and one third of newly diagnosed cases are already metastatic. Patients with metastatic RCC have a strikingly poor prognosis with a 5-year survival rate of <10%.^1^ Despite the advent of new targeted therapies, tumors frequently develop resistance to these therapies by activating bypass pathways.^2–4^ One of the major mechanisms of resistance is to induce the activation of upstream receptor tyrosine kinases, which subsequently stimulate signal-transduction cascades, leading to continued tumor growth. For instance, under the inhibition of mammalian target of rapamycin (mTOR), the insulin-like growth factor-1 receptor (IGF1R) is activated to upregulate its downstream signaling pathways, including 1) the PI3K (phosphoinositide-3-kinase)/AKT/mTOR pathway to support cancer cell survival and growth; 2) the Ras/ERK pathway to increase cell cycle progression and proliferation; and 3) Src/STAT3 (signal transducer and activator of transcription 3) signaling to induce epithelial-mesenchymal transition, cell motility, and invasiveness.^5–7^ In view of the importance of IGF1R in promoting oncogenesis, some IGF1R inhibitors, e.g., linsitinib (OSI-906), have been undergoing clinical investigations in advanced solid tumors and are considered potential next-line therapies.^8–11^ However, many issues remain to be resolved, especially the identification of patients who are unlikely to show early benefit from this therapy.

Metabolic pathways appear to be dysregulated in aggressive RCC, and complete cures of this disease are elusive. Both gain and loss of function in genes encoding key metabolic enzymes result in altered levels of metabolites and thereby promote tumor development and/or progression.^12,13^ To date, only a limited number of metabolic enzymes and metabolites with transforming properties have been identified in the context of tumors, and few of them relate to RCC. A recent integrated metabolomics analysis of the pathway alterations underlying RCC has uncovered dysregulated polyamine pathway associated with stage progression.^14^ Of note, a metabolite related to this pathway, methylthioadenosine (MTA), is significantly increased in aggressive RCC. MTA is the substrate of methylthioadenosine phosphorylase (MTAP), a catabolic enzyme that rescues adenine and methionine through the catalysis of MTA phosphorylation, which ultimately yields adenine and methylthioribose-1-phosphate.^15^ As expected, given the role of MTAP in MTA metabolism, significant MTA accumulation has been observed in MTAP-deficient cells, particularly in cancers.^16–19^

Many studies have reported a lack of MTAP in numerous human tumors, including melanoma, gliomas, hepatocellular carcinoma, and pancreatic, lung, breast, and blood-related cancers.^18,20–27^ However, the molecular mechanisms underlying MTAP-mediated tumor suppression have yet to be elucidated. Alarmingly, the function of MTAP in various cancers has been conflicting. In certain malignancies, MTAP does not act as a tumor suppressor; instead, MTAP inhibition slows the growth of human head and neck, prostate and lung cancers.^22,28,29^ Our understanding of MTAP in tumor biology is not complete, and there have been no studies thus far on the functional roles of MTAP in kidney cancer.

In this study, we first confirm the clinical significance and functionality of MTAP in RCC tissues and cells. Next, we demonstrate the mechanisms of MTAP-mediated tumor suppression via the utilization of a phospho-RTK antibody array screen and identify IGF1R as a driver pathway in MTAP-deficient RCC. The critical aim of this study is to facilitate the translation from in vitro discovery into pre-clinical and clinical trials for the treatment of advanced RCC.

## Results

### MTAP loss and/or downregulation contributes to RCC progression

**Fig. 1:**
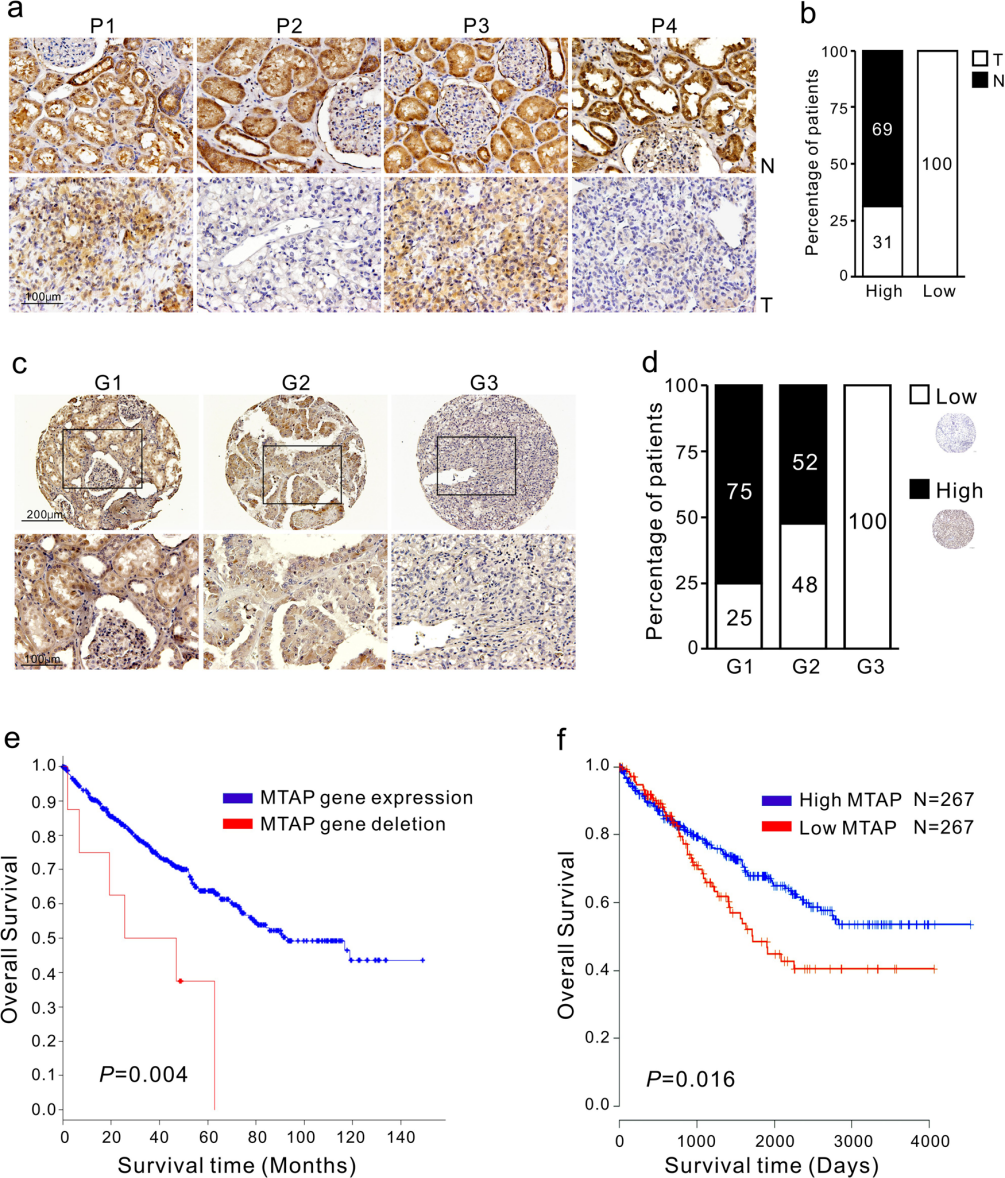
Clinical relevance of MTAP expression in RCC tumor grade and overall survival. **a** Low immunohistochemical (IHC) staining of MTAP protein in tumor (T) vs adjacent non-tumor areas (N) from RCC patients; P1, P2, P3 and P4 are four representative patients. **b** Percentage of patients with high and low MTAP according to tumor (T) vs adjacent non-tumor areas (N). Numbers in bars represent the percentage of patients for each condition. PANEL c and d: representative IHC images of grades 1, 2 and 3 (G1, G2, and G3) RCC tumors using an anti-MTAP antibody (*n* = 56). **c** An enlarged image from the dark frame is shown at the bottom. **d** Percentage of patients with high and low levels of MTAP expression corresponding to tumor grade. Numbers in bars represent the percentage of patients for each condition. **e**, **f** analysis of MTAP expression in The Cancer Genome Atlas (TCGA) dataset for RCC. **e** Patients were grouped into MTAP-expressed or MTAP-deleted status, and their overall survival rate was analyzed by Kaplan-Meier plot and two-sided log-rank tests (*n* = 538). **f** Patient samples were grouped into high and low MTAP expression categories. The survival of the two groups was plotted using the Kaplan–Meier method, and differences in survival were analyzed using two-sided log-rank tests (*n* = 534)

The MTA-related polyamine pathway was previously reported to correlate with RCC stage.^14^ To determine whether MTA is associated with RCC tumor grade, we analyzed MTA levels in RCC tissues with various grades from the same dataset^14^ and found a grade-dependent upregulation of MTA abundance (Supplementary Figure S1, *n* = 138). Because MTA accumulation is attributed to MTAP loss, we examined the expression pattern of MTAP in human RCC and adjacent normal tissue samples by immunohistochemistry (IHC) staining. The IHC results were classified into low- and high-expression categories according to the intensity and extent of staining, as described in “Materials and Methods”. We observed a decrease in MTAP protein expression in RCC tissues (T) compared to adjacent normal tissues (N) (Fig. 1a). Specifically, 56% (*n* = 28/50) of RCC tumors had low MTAP protein expression, while high MTAP expression was detected in 100% (*n* = 50/50) of adjacent normal parts (Fig. 1b, *p* < 0.0001). We next evaluated whether MTAP expression is associated with RCC tumor grade. In a screen of nephrectomy samples from another cohort of 56 patients with RCC, we found that abundant MTAP staining in samples was significantly greater in patients with grade 1 (G1) RCC than in patients with grade 2 (G2) and grade 3 (G3) RCC (Fig. 1c), at a rate of 75% versus 52 and 0%, respectively (Fig. 1d). MTAP staining was inversely correlated with tumor grade and gradually decreased in higher-grade RCC (Supplementary Table S1; *p* < 0.0001).

We next evaluated the association of MTAP gene expression with overall survival in RCC patients from The Cancer Genome Atlas (TCGA). Data from the cBioPortal analysis of the TCGA dataset for RCC (*n* = 538) showed that deletion in the MTAP gene was associated with a significant decrease in survival (Fig. 1e, *p* = 0.004). Patient samples (*n* = 534) were then grouped into low and high MTAP categories, and we demonstrated that patients with low MTAP expression had a significantly shorter overall survival than those with high MTAP expression (Fig. 1f, *p* = 0.016). Therefore, MTAP expression is likely of more importance in patients with low-grade RCC and a better prognosis.

### MTAP reverses epithelial-mesenchymal transition and inhibits cancer migration/invasion

**Fig. 2:**
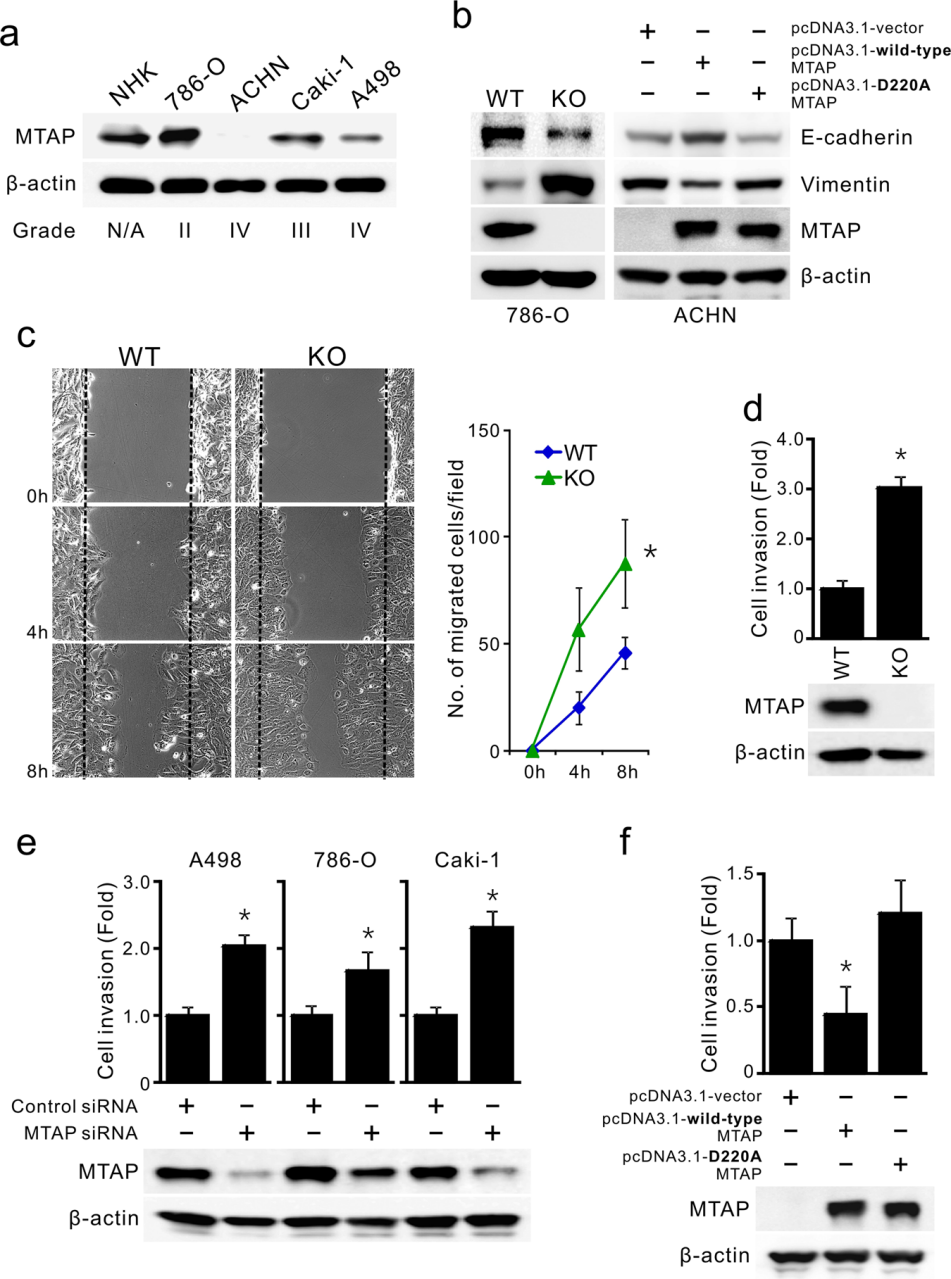
MTAP reduces the expression of mesenchymal markers and cancer cell migration and invasiveness. **a** MTAP protein levels in normal human kidney (NHK) cells and several RCC cell lines with various tumor grades were analyzed by Western blot assays. **b** Effect of MTAP expression on EMT markers. Cell lysates from 786-O cells with MTAP gene knockout or ACHN cells transiently overexpressing wild-type or D220A-mutated MTAP were subjected to immunoblotting with the indicated antibodies. **c** 786-O cells with (KO) or without (WT) MTAP gene knockout were subjected to scratch/wound-healing assays, and the number of cells migrating to the wound area was monitored (left) and quantified (right) at 0, 4 and 8 h post-scratching. *p < 0.05 vs WT (*n* = 3, mean ± SD). **d** Cell invasion abilities of 786-O WT and KO cells were determined by Matrigel transwell assays. *p < 0.05 vs WT (*n* = 3). **e** Multiple RCC cells as indicated were transfected with control or MTAP-specific siRNAs. After 72 h of transfection, cells were subjected to transwell invasion assays (top) and Western blot analyses (bottom). Data are expressed as the mean ± SD (*n* = 4), *p < 0.05 compared with cells receiving control siRNA. **f** Effects of ectopic V5-tagged wild-type or D220A-mutated MTAP expression on ACHN cell invasion by using transwell invasion assays (top, *n* = 4). The transfection efficiency was confirmed by Western blot (bottom)

Because of the inverse correlation between MTAP expression and RCC progression, we hypothesized that the metabolic enzyme MTAP plays an inhibitory role in the aggressive nature of kidney cancer. We assessed the MTAP abundance in primary normal human kidney epithelial (NHK) cells and several commercially available RCC cell lines and discovered reduced MTAP expression in high-grade RCC cell lines (e.g., Caki-1, A498, ACHN; Fig. 2a), consistent with the results in Figs. 1c–d. Next, we used a CRISPR/Cas9 approach to eliminate MTAP expression in low-grade RCC, 786-O cells, producing MTAP-knockout (KO) cells. MTAP-knockout RCC cells displayed an elongated, spindle-like morphology with extended pseudopodial branches (Supplementary Figure S2). We noticed an increase of the mesenchymal marker vimentin but a reduction of the epithelial marker E-cadherin in MTAP KO cells (Fig. 2b, left). To further confirm that epithelial-mesenchymal transition (EMT) results from MTAP deficiency, we ectopically overexpressed V5-tagged wild-type MTAP in MTAP gene–deficient ACHN cells, a high-grade RCC cell line. Figure 2b (right) shows that enforced expression of wild-type MTAP augmented E-cadherin levels and diminished vimentin expression in ACHN cells. Interestingly, these alterations were dramatically attenuated in cells transfected with an enzymatically defective MTAP, D220A mutant (Fig. 2b, right), implying that EMT inhibition is associated with MTAP enzymatic activity.

Because the EMT process is the acquisition of the ability to migrate and invade, we next examined the cell migration and invasion capabilities of MTAP KO cells. Using a scratch/wound-healing assay, we observed an increased number of migrated cells in response to MTAP loss (Fig. 2c). Likewise, MTAP KO cells displayed an elevated invasion ability compared to WT cells (Fig. 2d). After 72 h of MTAP expression knockdown using MTAP-specific siRNAs, cell invasiveness significantly increased in multiple RCC cell lines (Fig. 2e). Conversely, overexpression of wild-type MTAP for 48 h impaired the invasive phenotype, but enzyme-defective D220A-MTAP failed to impair this phenotype (Fig. 2f). Altogether, our results provide evidence for a critical role for MTAP in suppressing the aggressiveness of high-grade RCC cells.

### MTAP modulates the crosstalk between protein arginine methylation and tyrosine phosphorylation

**Fig. 3:**
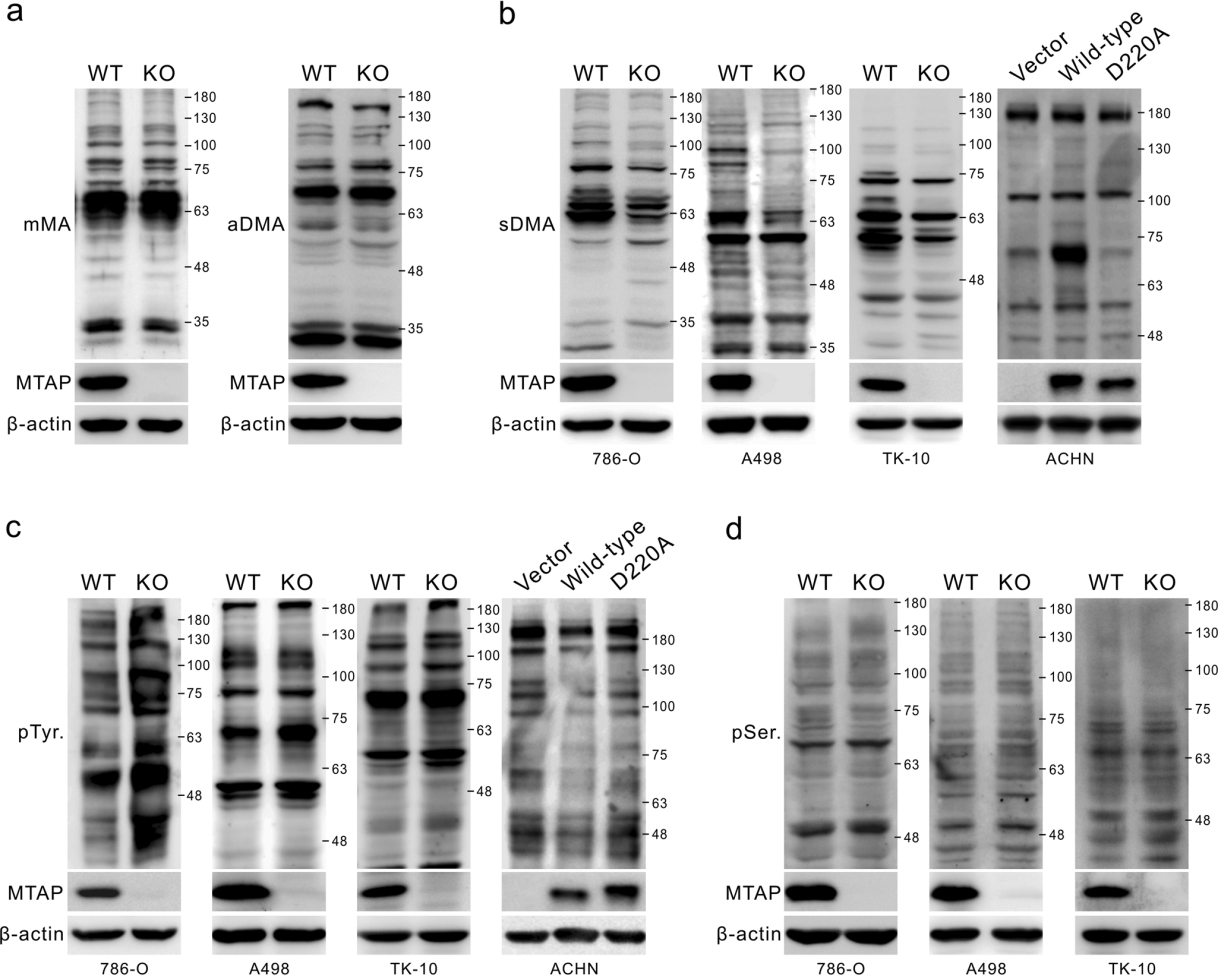
Changes in profiling of phospho- and symmetrically dimethyl-proteins in response to alteration of MTAP expression. **a**, **d** lysates from several MTAP-knockout RCC cells, including 786-O, A498 and TK-10 cells, and ACHN cells transiently overexpressing wild-type or D220A-mutated MTAP, were evaluated for the levels of mMA, aDMA (**a**) and sDMA (**b**), phospho-tyrosine (pTyr) (**c**), and phospho-serine (pSer) (**d**) by Western blots using antibodies as indicated

Cancer cells with MTAP loss exhibit a significant accumulation of MTA, which is not only a metabolic intermediate in the conversion of putrescine to spermidine and of spermidine to spermine^30^ but also a pharmacological agent used to inhibit arginine methylation.^16,17,25,31^ To determine the effect of MTAP expression on arginine methylation in RCC cells, we measured levels of monomethylarginine (mMA), asymmetric dimethylarginine (aDMA) and symmetric dimethylarginine (sDMA) in both MTAP-expressing (WT) and MTAP KO cells. Western blots showed no changes in total mMA and aDMA in MTAP KO cells compared to MTAP-expressing cells (Fig. 3a and Supplementary Figure S3a). However, we observed that the levels of sDMA in MTAP KO 786-O cells were remarkably lower than those in WT 786-O cells (Fig. 3b, left). This phenomenon was recapitulated in A498 and TK-10 RCC cell lines as well upon MTAP deletion (Fig. 3b, middle). In addition, compared to that in cells transfected with the mock or D220A-mutated MTAP construct, we noted an increase in sDMA in ACHN cells with the ectopic expression of V5-tagged wild-type MTAP to metabolize MTA (Fig. 3b, right).

Recent studies have revealed crosstalk between protein post-translational modifications, such as methylation and phosphorylation. Because an interplay between methylation and phosphorylation is known to be an important mechanism of the regulation of receptor tyrosine kinase signaling,^31–36^ we analyzed overall protein tyrosine phosphorylation levels in the context of MTAP expression. Intriguingly, the levels of protein tyrosine phosphorylation were increased in multiple MTAP KO cells (Fig. 3c). As expected, wild-type MTAP downregulated protein tyrosine phosphorylation levels in ACHN cells, but D220A mutant MTAP did not. We also examined overall protein serine phosphorylation levels in various RCC cell lines upon MTAP loss, but these levels were unaltered upon MTAP knockout (Fig. 3d and Supplementary Figure S3b).

### Loss of MTAP expression activates IGF1R and its downstream signaling

**Fig. 4:**
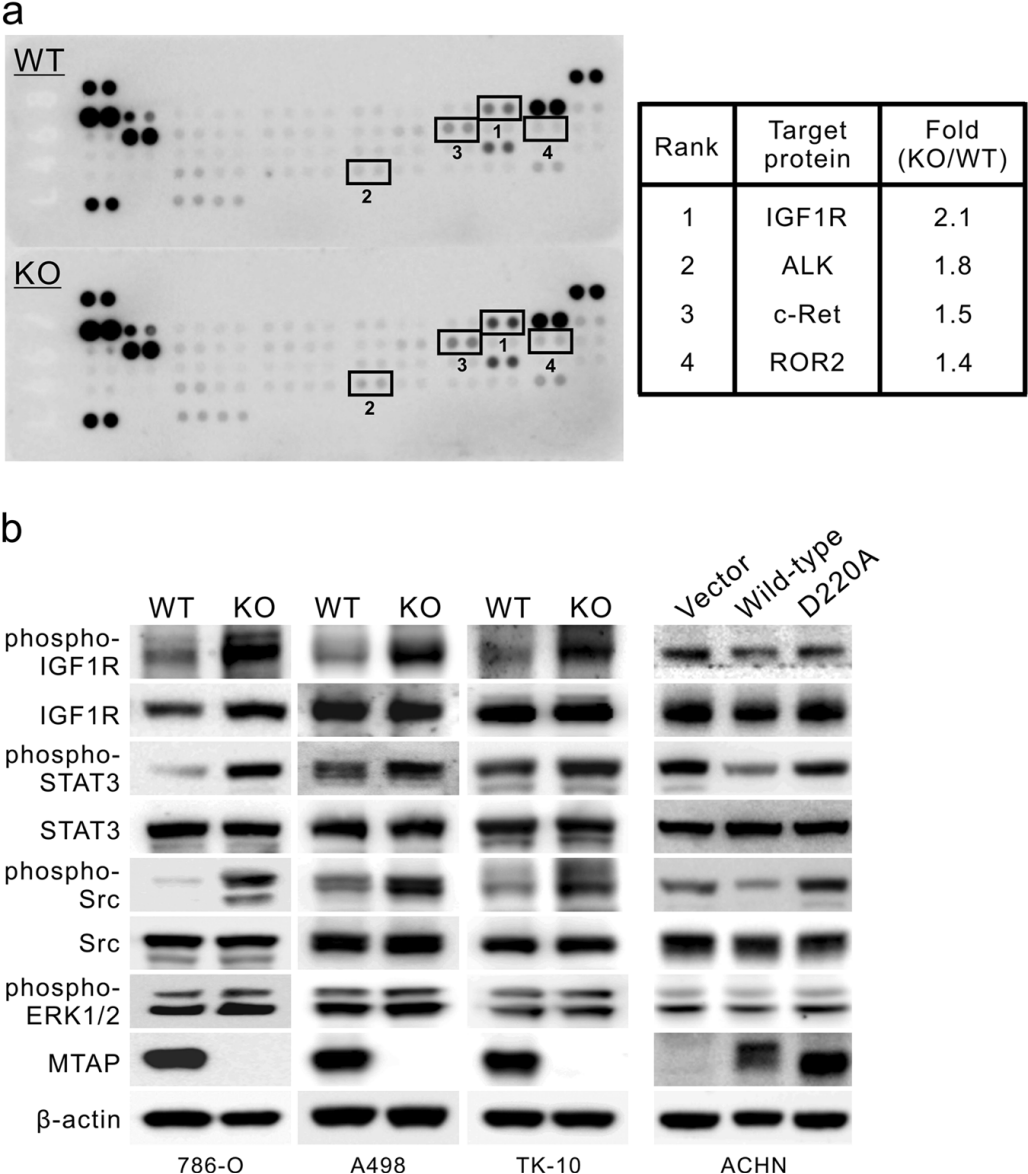
IGF1R activity is associated with MTAP deficiency. **a** Human Phospho-Receptor Tyrosine Kinase (RTK) Array blots. The activated RTKs were determined in 786-O cells with (KO) or without (WT) MTAP knockout. Lysates from MTAP WT and KO cells of 786-O were subjected to human phospho-receptor tyrosine kinase (RTK) arrays (left). The top four upregulated phospho-RTKs in MTAP-knockout cells are shown in the right panel (KO versus WT). **b** Immunoblotting analysis of the IGF1R autophosphorylation level and its downstream activities of STAT3, Src and ERK1/2 in multiple MTAP-knockout cells, including 786-O, A498, and TK-10 cells and V5-tagged MTAP-overexpressing ACHN cells

The tumor-suppressive function of MTAP in RCC has been established, but its molecular contribution has yet to be elucidated. Given that tyrosine phosphorylation is altered after MTAP knockout (Fig. 3c), we performed a phospho-receptor tyrosine kinase (RTK) array screen to identify potential RTKs regulated by MTAP expression. Fig. 4a lists the four top-ranked RTKs with respect to tyrosine phosphorylation upregulation in response to MTAP loss. Type 1 insulin-like growth factor-1 receptor (IGF1R) was the top candidate, with a 2.1-fold increase in MTAP-deleted cells (Fig. 4a, right) compared to MTAP WT cells. IGF1R also drew our attention because of its importance in RCC.^6^

To validate the regulation of IGF1R and its downstream signaling by MTAP expression, lysates from MTAP-knockout and MTAP-overexpressing cell lines were subjected to Western blots. An elevation of IGF1R phosphorylation at Tyr1131, the major autophosphorylation site, was observed in various RCC cells lacking MTAP (Fig. 4b, left), whereas inhibition of IGF1R activity was observed in ACHN cells overexpressing V5-tagged wild-type MTAP but not in cells transfected with D220A-mutated MTAP (Fig. 4b, right). In addition to IGF1R autophosphorylation, the activities of its downstream molecules involved in RCC aggressiveness, including STAT3, Src, and ERK1/2, were also negatively associated with MTAP expression level, supporting the notion that MTAP expression regulates IGF1R activity and its signaling pathways.

Because the crosstalk between protein methylation and phosphorylation is an important regulatory mechanism by which receptor tyrosine kinases are activated and trigger cell signaling^32^ and in light of the fact that the downregulation of protein-methylation levels acts in parallel with increased tyrosine phosphorylation upon MTAP alteration, we suspected that tyrosine phosphorylation of IGF1R is modulated by its arginine methylation. Through immunoprecipitation of methylated arginine using an anti-sDMA antibody, we found an impaired level of symmetric arginine dimethylation on IGF1R protein precipitated from multiple MTAP-knockout cells with higher IGF1R autophosphorylation, suggesting the possibility that MTAP-mediated alteration in arginine methylation regulates IGF1R activity (Supplementary Figure S3c).

### MTAP deletion-mediated activation of IGF1R signaling is repressed by linsitinib

**Fig. 5:**
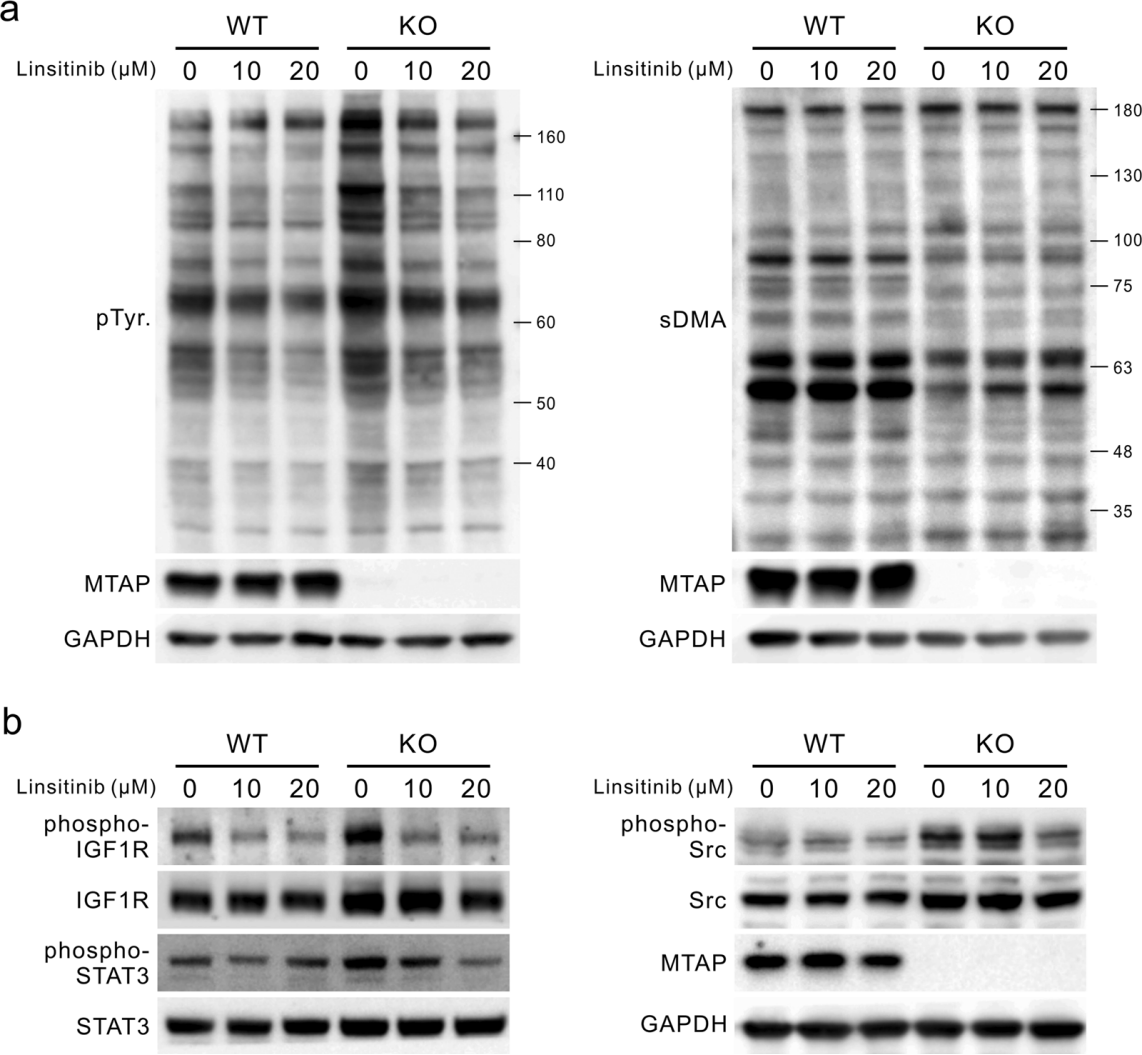
IGF1R inhibition reverses the oncogenic signaling induced by MTAP loss. **a** MTAP-knockout (KO) and wild type (WT) 786-O cells were treated with various doses of IGF1R inhibitor linsitinib for 24 h and analyzed for phospho-tyrosine (pTyr, left) and sDMA (right) levels by immunoblotting. **b** IGF1R activity and its downstream signaling molecules in WT and MTAP-KO 786-O cells were evaluated by Western blot assays after exposure to linsitinib for 24 h

Upregulation of IGF1R activity is capable of promoting EMT, cell invasiveness, and motility through positively modulating numerous signaling effectors, e.g., Src and STAT3.^6^ Given an increase in IGF1R autophosphorylation upon MTAP loss, we treated RCC cells with the selective IGF1R inhibitor linsitinib (OSI-906) to pharmacologically block IGF1R autophosphorylation and activation of the downstream signaling proteins.^11^ As shown in Fig. 5a (left), MTAP KO cells displayed higher levels of protein tyrosine phosphorylation than did WT 786-O cells. After treatment with linsitinib for 24 h, both cell lines exhibited suppressed protein tyrosine phosphorylation. Protein tyrosine phosphorylation levels in MTAP KO cells appeared to be similar to those in WT 786-O cells in the presence of linsitinib. The suppression of protein tyrosine phosphorylation was obvious in MTAP KO cells, suggesting that IGF1R inhibition is more effective in MTAP-deficient cells. Additionally, we confirmed that sDMA levels in both groups were not affected by linsitinib treatment to prove that suppression of protein tyrosine phosphorylation is directly caused by IGF1R inhibition, not a change in sDMA levels (Fig. 5a, right). Fig. 5b shows the activities of IGF1R and its downstream molecules upon exposure to linsitinib. The level of IGF1R autophosphorylation at Tyr1131 decreased concurrently with the reduced phosphorylation of both STAT3 at Tyr705 and Src at Tyr416 after 24 h of linsitinib treatment. This inhibitory effect was particularly evident in MTAP KO cells, suggesting that IGF1R becomes a key player in regulating signaling molecules in the absence of MTAP expression.

### IGF1R inhibition attenuates the malignant phenotypes of MTAP-deficient RCC

**Fig. 6:**
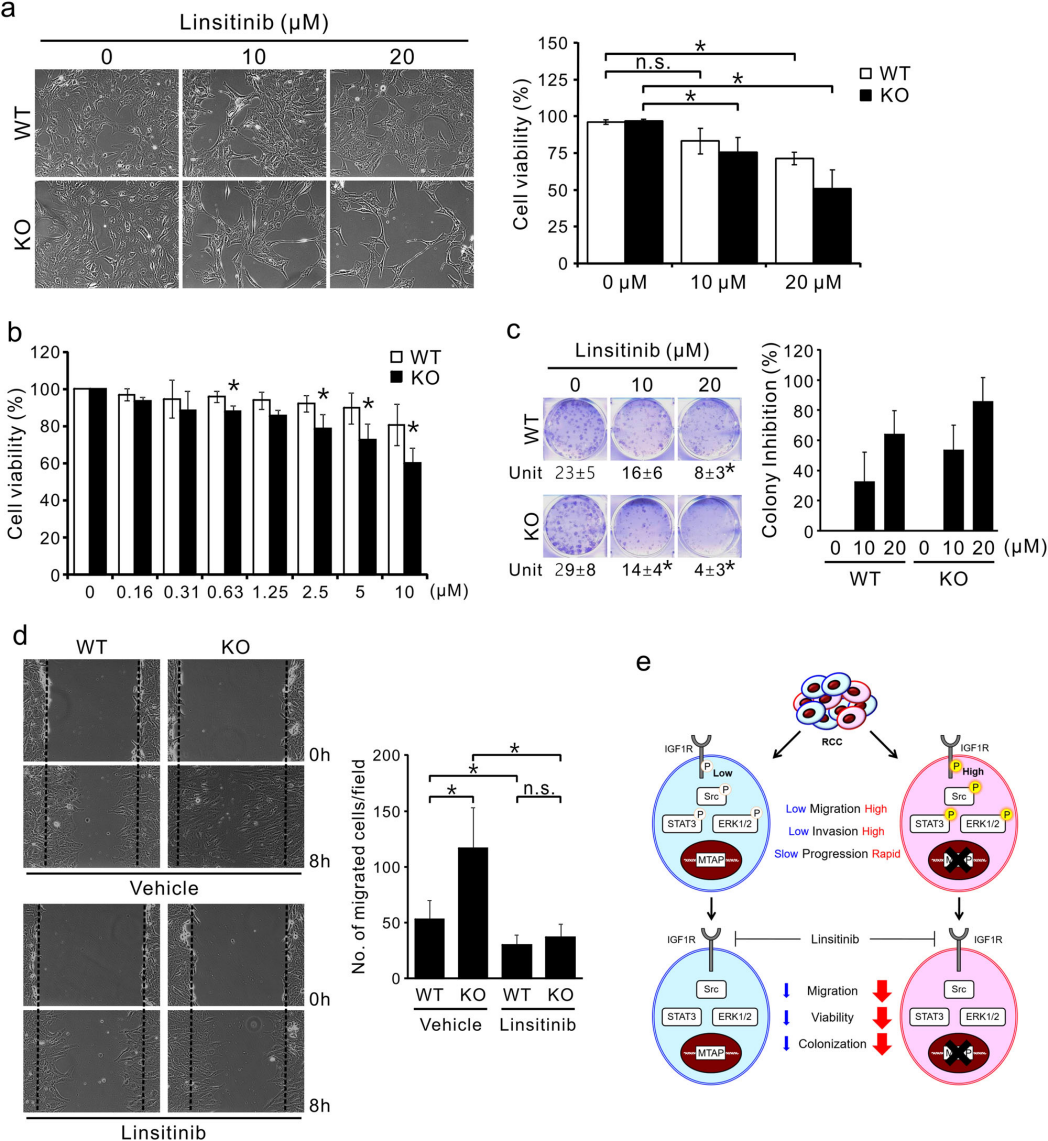
Linsitinib treatment impairs cancer-promoting effects resulting from MTAP loss. **a** Effects of linsitinib treatment on the cell growth and viability of wild type (WT) and MTAP-knockout (KO) 786-O cells were determined by trypan blue exclusion assays. After 72 h of treatment, cell confluency was microscopically photographed (left), and cells were collected for the trypan blue exclusion assay (right). *p < 0.05 (*n* = 4, mean ± SD). **b** Cell viability of wild type (WT) and MTAP-knockout (KO) 786-O cells after 72 h of treatment with numerous concentrations of linsitinib was measured by MTT assays. *p < 0.05 compared to WT (*n* = 6, mean ± SD). **c** Cells were exposed to two concentrations of linsitinib, and colonies were counted after 8 days using crystal violet staining. Left (top), representative pictures of five independent experiments are shown. Left (bottom), number of colony units (mean ± SD). *p < 0.05 vs untreated cells (0 μM). Right, the degree of colony inhibition relative to untreated cells (0 μM). **d** Confluent cultures of wild type (WT) and MTAP-knockout (KO) 786-O cells were treated with 10 μM linsitinib for 24 h and then subjected to scratch/wound-healing assays. Cells were monitored microscopically at 0 and 8 h after scratching. Left, representative phase contrast pictures. Right, quantification of the numbers of cells migrated to the wound area after scratching. Data shown as the mean ± SD; *p < 0.05 (*n* = 9). **e** Proposed hypothetical models for the mechanism of MTAP-mediated suppression of RCC cell invasion and migration

Based on the above results showing that a lack of MTAP contributes to RCC aggressiveness and activation of IGF1R signaling and in view of the critical roles of IGF1R in RCC oncogenesis,^6^ we attempted to test whether inhibition of IGF1R activity by linsitinib treatment impairs oncogenic phenotypes in MTAP-deficient cells. Both MTAP KO and WT 786-O cells were exposed to two different doses of linsitinib for 48 h. Cell confluency in both groups was reduced in a dose-dependent manner, although cell number reduction was greater in the MTAP KO group (Fig. 6a, left). Our trypan blue exclusion tests showed that exposure to 10 μM linsitinib led to a significant decrease in cell viability in MTAP KO cells; however, the effective concentration for WT 786-O cells was 20 μM (Fig. 6a, right). To determine whether MTAP KO cells are more sensitive than WT cells to linsitinib, cells were exposed to increasing concentrations of linsitinib for 72 h, and cell viability was evaluated by MTT assays. We showed that linsitinib-mediated cytotoxicity is increased in MTAP KO cells and that the IC_50_ (half-maximal inhibitory concentration) of linsitinib decreases from 27.38 to 11.23 μM after the knockout of MTAP (Fig. 6b).

In addition to suppressing cell viability, linsitinib treatment also inhibited the colony-forming ability in both types of RCC cells, and this inhibition appeared to be much stronger in MTAP KO cells versus WT 786-O cells (Fig. 6c). To determine whether linsitinib reduces RCC cell motility, cells were incubated with linsitinib for 24 h and subjected to wound-healing assays. As expected, a 3.2-fold reduction in the number of migrated cells was observed in the MTAP KO group after linsitinib treatment, while IGF1R inhibition decreased the cell migration capability of WT 786-O cells by 1.8-fold (Fig. 6d). These data convincingly demonstrate the requirement for IGF1R activity for the maintenance of aggressive phenotypes in MTAP-deleted cells.

## Discussion

RCC has emerged as a metabolic disease characterized by dysregulated expression of metabolic enzymes and altered levels of metabolites.^13^ Patients with metastatic RCC have an unusually poor prognosis and low response or near-universal resistance to all current therapies. To improve RCC treatment and the survival rate of patients with RCC, there is an urgent need to reveal the mechanisms by which metabolic enzymes and aberrant pathways regulate oncogenic signaling and/or to discover potential metabolites useful for predicting malignant changes leading to RCC. In this study, we identified dysregulated expression of the metabolic enzyme MTAP in aggressive RCC and demonstrated that MTAP-mediated RCC suppression occurs because of inhibited IGF1R signaling.

The *MTAP* gene is located on chromosome 9p21 and is frequently found to be co-deleted with *CDKN2A* and *CDKN2B*, which encode p16 and p15, respectively.^37,38^ Given that p16 and p15 are thought to be tumor suppressors and their loss facilitates cell cycle progression,^39,40^ it is unclear whether the clinical effect of MTAP deletion occurs independently of these events or is an incidental consequence. A handful of clinical studies have found deletions of the *MTAP* gene without concordant loss of *CDKN2A* or *CDKN2B*, and its deletion rate is higher than that of *CDKN2A* in certain cancers.^18,23^ In this study, we verified an indispensable role of MTAP loss in RCC progression. In our clinical observations, we show that a significant percentage of RCC tumors have low MTAP expression and that MTAP expression is inversely associated with tumor grade and shortens patient survival. Consistent with other cancers,^20,25,27^ our bio-functional assays prove that MTAP plays an inhibitory role in oncogenic progression, particularly in cell motility and invasion. These results attest to the contribution of MTAP to RCC suppression and the potential usage of MTAP as a marker in predicting malignant behavior in RCC patients.

Only a limited number of putative oncometabolites with transforming properties have been identified thus far in the context of tumors, and most of them are involved in the tricarboxylic acid cycle.^41^ Since accumulated oncometabolites can be easily detected in the body fluids of patients, discovering novel oncometabolites for predicting the prognosis and malignant biological behavior is a reasonable line of investigation. Our study reveals that MTA may be a potential oncometabolite associated with an aggressive nature in RCC. Several reports have indicated a specific contribution of MTA to different cell types in the tumor microenvironment. MTA administration to increase cellular MTA levels results in the upregulation of matrix metalloproteinases and growth factors in melanoma cells, hepatocellular carcinoma cells, and fibroblasts.^25,42^ Moreover, accumulated MTA was found to repress T-cell proliferation, activation, and differentiation.^43^ Despite these observations, future studies on the targeting of the MTAP/MTA axis must prioritize investigating the mechanisms underlying MTA regulation in neoplastic disease and its role in the context of MTAP deficiency.

The catalysis of MTA phosphorylation by MTAP is necessary for cells to carry out polyamine metabolism. Many cancer cells exhibit a loss of MTAP expression, which contributes to significant MTA accumulation.^16–19^ In addition to a metabolic intermediate in the conversion of putrescine to spermidine and of spermidine to spermine,^30^ MTA serves as a potent and selective inhibitor of the protein arginine methyltransferase family (PRMT), including type I (e.g., PRMT1) and type II (e.g., PRMT5) PRMTs.^16,17,25,31^ In arginine methylation, PRMTs transfer methyl groups to the guanidine nitrogen of specific arginine residues on their target proteins, and this methylation alters signal transduction and cellular functions. Both type I and type II PRMTs generate monomethylarginine (mMA) as an intermediate; type I PRMTs further catalyze the formation of asymmetric dimethylarginine (aDMA), and type II PRMTs catalyze the generation of symmetric dimethylarginine (sDMA).^32^ MTA was found to be favorable to the inhibition of PRMT5 activity.^16,17,19^ Here, we showed that various MTAP-deleted RCC cells exhibit a reduction in sDMA levels.

sDMA modifications of target proteins may lead to changes in protein structure, localization, activity, interaction with other proteins, or intramolecular posttranslational modification crosstalk.^32^ sDMA modification of non-histone proteins and histones plays a crucial role in modulating cellular processes. Of most interest, protein phosphorylation due to sDMA modification is an important regulatory mechanism in receptor tyrosine kinase signaling and tumorigenesis.^31–36^ For instance, arginine methylation on the epidermal growth factor receptor alters its tyrosine phosphorylation level, thereby modulating carcinogenesis, therapy response and recurrence.^36,44^ In a simultaneous examination of both protein methylation and phosphorylation, we observed a decrease in protein methylation levels concomitant with an increase in tyrosine phosphorylation in MTAP-deleted cells, suggesting an interplay between sDMA and phosphotyrosine regulated by MTAP/MTA abundance. Identifying arginine-methylation and tyrosine-phosphorylation signatures altered by MTAP expression is an important line of investigation for future studies.

Based on the current evidence showing an upregulation of phosphotyrosine levels in response to MTAP loss, we used phospho-RTK antibody arrays to reveal that a lack of MTAP results in elevated levels of IGF1R autophosphorylation at Tyr1131. IGF1R has been recognized as a driver of malignant transformation, and its activation and overexpression are associated with poor survival in RCC,^6^ making it an attractive candidate for further study. The canonical activation of IGF1R is stimulated by its ligand IGF-1, leading to the induction of downstream signaling cascades.^6^ Using a CRISPR/Cas9 approach to knockout MTAP and ectopic expression of wild-type and D220A-mutated MTAP, we found that both IGF1R activity and its downstream pathways, especially Src and STAT3 signaling, are regulated by MTAP abundance and its enzyme activity. However, further studies are needed to determine whether this non-canonical activation of IGF1R is associated with sDMA modification.

Because of the importance of the IGF1R pathway in cancers, various inhibitors, including monoclonal antibodies and small molecules targeting IGF1R or IGF-1, have been developed, most of which are undergoing clinical trials.^45^ To the best of our knowledge, this is the first report to establish the signaling axis of MTAP-IGF1R. First, our data offer insights into various signaling networks occurring in kidney cancer and can show how different pathways intersect and interact. Second, our results link metabolic pathways, post-translational modifications, and signal transduction pathways to the stringent regulation of RCC progression. Third, our study presents a promising molecular model and provides an understanding of how aggressive RCC cells can shift from using metabolic pathways to signal transduction pathways, thereby providing a growth advantage to tumor cells.

MTAP deletion in certain cancers has been observed for decades, but treatment for these patients has had limited success in the clinic. Although PRMT5 inhibitors are a plausible approach, in vitro studies using the synthetic lethal model built from genome-scale pooled shRNA screening showed only moderate efficacy.^16^ Inhibition of de novo purine synthesis or methionine deprivation are promising strategies given that MTAP-deleted cells are unable to use salvage pathways.^46^ Combinational treatment with MTA and chemotherapeutic agents (6-thioguanine, 5-fluorouracil, or 2-fluoroadenine) seems to selectively protect MTAP-intact cells and kill MTAP-deficient tumors.^47,48^ Unfortunately, these strategies are still under in vitro and in vivo tests. Our current work has verified that MTAP-deficient cells require IGF1R activity and that IGF1R signaling may be a driver pathway that supports RCC cell survival and malignancy. Thus, the identification of IGF1R’s contribution to MTAP-deleted cells provides another therapeutic approach: suppression of IGF1R signaling to target RCC cells with MTAP downregulation. This study represents the first investigation into the use of an IGF1R inhibitor in treating aggressive RCC. Blockage of IGF1R signaling in MTAP-deleted RCC by linsitinib efficiently reverses its oncogenic phenotype. Notably, this therapeutic application also demonstrates that a certain population of patients might respond well to linsitinib, which has been stuck in phase II/III clinical trials.^8–10^

In summary, we provide evidence that MTAP deficiency activates IGF1R signaling, thereby promoting RCC progression. The use of linsitinib to block IGF1R-mediated functions reverses oncogenic characteristics caused by MTAP loss and/or downregulation (Fig. 6e). Importantly, our studies contribute to a better understanding of how metabolic enzymes participate in the regulation of post-translational modifications and cancer progression, allowing for the development of novel targeted therapies and potential therapeutic strategies for RCC.

## Materials and methods

### Materials, cell culture, and transfection

All reagents, primers and antibodies used in this study as well as detailed information on cell culture and transfection are described in the Supplementary Methods.

### Patient tumor specimens and immunohistochemical staining

Kidney tumors from 56 patients were obtained from patients with histologically confirmed RCC who underwent surgical resection at the UC Davis Comprehensive Cancer Center (Sacramento, CA) after approval by the Institutional Review Board of the UC Davis Health System. Written informed consent was obtained from all patients. Formalin-fixed and paraffin-embedded specimens were used, and immunohistochemical staining was performed for MTAP expression as described previously.^49,50^ Detailed information on staining and scoring is found in the Supplementary Methods.

### Human phospho-receptor tyrosine kinase (RTK) array

The Human Phospho-RTK Array Kit (# ARY001B) was purchased from R&D Systems (Minneapolis, MN). We performed array screening according to the manufacturer’s protocol. Briefly, cell lysates were incubated with the phospho-RTK array membranes. After washing, the membranes were incubated with biotinylated antibody cocktail. The amounts of phospho-RTK were assessed with streptavidin conjugated to horseradish peroxidase, followed by chemiluminescence detection. The density of each dot was quantified against the average of the internal controls on the membrane as indicated in the protocol.

### Cell invasion assay

In vitro cell invasion assays were performed as previously described^49^ using transwell chambers (8 μm pore size; Costar, Cambridge, MA). Filters were coated with Matrigel (Becton Dickinson, Franklin Lakes, NJ), and 2 × 10^4^ cells were seeded onto the Matrigel. After 20 h of incubation, filters were swabbed with a cotton swab, fixed with methanol and then stained with Giemsa solution (Sigma, St Louis, MO). The cells attached to the lower surface of the filter were counted under a light microscope.

### Scratch/wound-healing assay

Cells were seeded into 6-well tissue culture dishes and grown to 90% confluence. Each confluent monolayer was then wounded linearly using a pipette tip and washed three times with PBS. Thereafter, cell morphology and migration were observed and photographed within 8 h. The number of cells migrating into the cell-free zone was acquired under a light microscope.

### Cell viability and colony formation assays

Trypan blue exclusion tests and MTT assays were used to quantitate viable cell numbers. For trypan blue tests, cells were plated on 12-well plates, grown to 75% confluence, and treated with the indicated concentrations of linsitinib. After 72 h, both attached and detached cells were collected and then stained with 0.2% trypan blue (0.1% final concentration), and the numbers of trypan blue-positive and -negative cells were counted using a hemocytometer under low-power microscopy. For MTT assays, cells were seeded into 96-well plates, grown to 75% confluence and cultured according to the indicated treatment. Cell viability was evaluated by the MTT assay according to the manufacturer’s protocol (Promega, Madison, WI). The absorbance at 570 nm was measured on a multi-well scanning spectrophotometer (Victor3; Perkin-Elmer, Boston, MA). For anchorage-dependent growth assays, 200 cells were seeded into each well of 6-well plates. Cells were treated with linsitinib at the indicated concentrations for 4 days and then changed to complete culture medium; these cells were further incubated for 4 days. Colonies were stained using 0.001% crystal violet, and the number of colonies with a diameter greater than 0.5 mm was counted under an inverted microscope.

### Western blot analysis and immunoprecipitation assays

Western blot analyses and the preparations of whole-cell lysates have been previously described.^49^ Whole cell lysates were prepared by lysing cells in lysis buffer (50 mM Tris-HCl (pH 7.4), 1% NP-40, 150 mM NaCl, 1 mM EDTA, 20 μg/ml leupeptin, 1 mM PMSF and 20 μg/ml aprotinin), and proteins were then separated by SDS-PAGE. Immunoblotting was conducted with appropriate antibodies followed by chemiluminescent detection. For immunoprecipitation analyses, whole cell lysates were cleaned by pre-incubation with Protein A/G PLUS-Agarose beads to remove non-specifically bound proteins. After precipitation with the appropriate antibodies and Protein A/G PLUS-Agarose beads, the immunoprecipitated complexes were washed, separated by SDS-PAGE and followed by Western blot assays.

### Statistical analysis

Data are presented as the mean ± SD of at least three independent experiments. The quantitative in vitro and in vivo data were analyzed using the Student’s *t*-test. The difference in patient characteristics between the high-expression and low-expression groups was examined using Fisher’s exact test. MTAP mRNA expression in samples from the TCGA dataset was examined using the Wilcoxon–Mann–Whitney test. In survival analyses, overall survival curves for groups with low versus high MTAP levels were plotted using the Kaplan-Meier method, and the differences in survival between high-level and low-level patients were analyzed using the log-rank test. All analyses were performed using SPSS software (v20.0; SPSS, Inc., Chicago, IL) and R Programming (Johns Hopkins University, Baltimore, MD). All statistical tests were two-sided, and *p* values < 0.05 were considered statistically significant.

## Supplementary information

Supplemental Material_clean

## References

[1] Siegel RL, Miller KD, Jemal A (2018). Cancer statistics, 2018. CA..

[2] Joosten SC (2015). Resistance to sunitinib in renal cell carcinoma: from molecular mechanisms to predictive markers and future perspectives. Biochim. Biophys. Acta.

[3] Santoni M (2014). Emerging strategies to overcome the resistance to current mTOR inhibitors in renal cell carcinoma. Biochim. Biophys. Acta.

[4] Zhou L (2016). Targeting MET and AXL overcomes resistance to sunitinib therapy in renal cell carcinoma. Oncogene.

[5] Wan X, Harkavy B, Shen N, Grohar P, Helman LJ (2007). Rapamycin induces feedback activation of Akt signaling through an IGF-1R-dependent mechanism. Oncogene.

[6] Tracz AF, Szczylik C, Porta C, Czarnecka AM (2016). Insulin-like growth factor-1 signaling in renal cell carcinoma. BMC. Cancer.

[7] Denduluri SK (2015). Insulin-like growth factor (IGF) signaling in tumorigenesis and the development of cancer drug resistance. Genes Dis..

[8] Ciuleanu TE (2017). Randomised Phase 2 study of maintenance linsitinib (OSI-906) in combination with erlotinib compared with placebo plus erlotinib after platinum-based chemotherapy in patients with advanced non-small cell lung cancer. Br. J. Cancer.

[9] Barata P (2018). A phase 2 study of OSI-906 (linsitinib, an insulin-like growth factor receptor-1 inhibitor) in patients with asymptomatic or mildly symptomatic (non-opioid requiring) metastatic castrate resistant prostate cancer (CRPC). Invest. New Drugs.

[10] Oza A (2018). Phase 2 study evaluating intermittent and continuous linsitinib and weekly paclitaxel in patients with recurrent platinum resistant ovarian epithelial cancer. Gynecol. Oncol..

[11] Mulvihill MJ (2009). Discovery of OSI-906: a selective and orally efficacious dual inhibitor of the IGF-1 receptor and insulin receptor. Future Med. Chem..

[12] Coller HA (2014). Is cancer a metabolic disease?. Am. J. Pathol..

[13] Wettersten HI (2015). Grade-dependent metabolic reprogramming in kidney cancer revealed by combined proteomics and metabolomics analysis. Cancer Res..

[14] Hakimi AA (2016). An integrated metabolic atlas of clear cell renal cell carcinoma. Cancer Cell..

[15] Avila MA, Garcia-Trevijano ER, Lu SC, Corrales FJ, Mato JM (2004). Methylthioadenosine. Int. J. Biochem. Cell. Biol..

[16] Kryukov GV (2016). MTAP deletion confers enhanced dependency on the PRMT5 arginine methyltransferase in cancer cells. Science.

[17] Marjon K (2016). MTAP deletions in cancer create vulnerability to targeting of the MAT2A/PRMT5/RIOK1 axis. Cell Rep..

[18] Woollard WJ (2016). Independent loss of methylthioadenosine phosphorylase (MTAP) in primary cutaneous T-cell lymphoma. J. Invest. Dermatol..

[19] Mavrakis KJ (2016). Disordered methionine metabolism in MTAP/CDKN2A-deleted cancers leads to dependence on PRMT5. Science.

[20] Christopher SA, Diegelman P, Porter CW, Kruger WD (2002). Methylthioadenosine phosphorylase, a gene frequently codeleted withp16(cdkN2a/ARF), acts as a tumor suppressor in a breast cancer cell line. Cancer Res..

[21] Hustinx SR (2005). Homozygous deletion of the MTAP gene in invasive adenocarcinoma of the pancreas and in periampullary cancer: a potential new target for therapy. Cancer Biol. Ther..

[22] Basu I (2011). Growth and metastases of human lung cancer are inhibited in mouse xenografts by a transition state analogue of 5’-methylthioadenosine phosphorylase. J. Biol. Chem..

[23] Su CY (2014). MTAP is an independent prognosis marker and the concordant loss of MTAP and p16 expression predicts short survival in non-small cell lung cancer patients. Eur. J. Surg. Oncol..

[24] Nobori T (1991). Absence of methylthioadenosine phosphorylase in human gliomas. Cancer Res..

[25] Kirovski G (2011). Down-regulation of methylthioadenosine phosphorylase (MTAP) induces progression of hepatocellular carcinoma via accumulation of 5’-deoxy-5’-methylthioadenosine (MTA). Am. J. Pathol..

[26] Gambichler T, Scola N, Bechara FG (2012). Significantly decreased methylthioadenosine phosphorylase expression in malignant melanoma. Am. J. Dermatopathol..

[27] Marce S (2006). Lack of methylthioadenosine phosphorylase expression in mantle cell lymphoma is associated with shorter survival: implications for a potential targeted therapy. Clin. Cancer Res..

[28] Basu I (2007). A transition state analogue of 5’-methylthioadenosine phosphorylase induces apoptosis in head and neck cancers. J. Biol. Chem..

[29] Chattopadhyay S, Zhao R, Tsai E, Schramm VL, Goldman ID (2006). The effect of a novel transition state inhibitor of methylthioadenosine phosphorylase on pemetrexed activity. Mol. Cancer Ther..

[30] Williams-Ashman HG, Seidenfeld J, Galletti P (1982). Trends in the biochemical pharmacology of 5’-deoxy-5’-methylthioadenosine. Biochem. Pharmacol..

[31] Mowen KA (2001). Arginine methylation of STAT1 modulates IFNalpha/beta-induced transcription. Cell.

[32] Biggar KK, Li SS (2015). Non-histone protein methylation as a regulator of cellular signalling and function. Nat. Rev. Mol. Cell Biol..

[33] Chen M (2016). Cross-talk between Arg methylation and Ser phosphorylation modulates apoptosis signal-regulating kinase 1 activation in endothelial cells. Mol. Biol. Cell..

[34] Boriack-Sjodin PA, Swinger KK (2016). Protein methyltransferases: a distinct, diverse, and dynamic family of enzymes. Biochemistry.

[35] Epstein DM, Buck E (2015). Old dog, new tricks: extracellular domain arginine methylation regulates EGFR function. J. Clin. Invest..

[36] Hsu JM (2011). Crosstalk between Arg 1175 methylation and Tyr 1173 phosphorylation negatively modulates EGFR-mediated ERK activation. Nat. Cell Biol..

[37] Illei PB, Rusch VW, Zakowski MF, Ladanyi M (2003). Homozygous deletion of CDKN2A and codeletion of the methylthioadenosine phosphorylase gene in the majority of pleural mesotheliomas. Clin. Cancer Res..

[38] Powell EL (2005). Concordant loss of MTAP and p16/CDKN2A expression in gastroesophageal carcinogenesis: evidence of homozygous deletion in esophageal noninvasive precursor lesions and therapeutic implications. Am. J. Surg. Pathol..

[39] LaPak KM, Burd CE (2014). The molecular balancing act ofp16(INK4a) in cancer and aging. Mol. Cancer Res..

[40] De Braekeleer M, Douet-Guilbert N, De Braekeleer E (2017). Prognostic impact of p15 gene aberrations in acute leukemia. Leuk. Lymphoma.

[41] Collins RRJ, Patel K, Putnam WC, Kapur P, Rakheja D (2017). Oncometabolites: a new paradigm for oncology, metabolism, and the clinical laboratory. Clin. Chem..

[42] Stevens AP (2009). Direct and tumor microenvironment mediated influences of 5’-deoxy-5’-(methylthio)adenosine on tumor progression of malignant melanoma. J. Cell. Biochem..

[43] Henrich FC (2016). Suppressive effects of tumor cell-derived 5’-deoxy-5’-methylthioadenosine on human T cells. Oncoimmunology.

[44] Liao HW (2015). PRMT1-mediated methylation of the EGF receptor regulates signaling and cetuximab response. J. Clin. Invest..

[45] Iams WT, Lovly CM (2015). Molecular pathways: clinical applications and future direction of insulin-like growth factor-1 receptor pathway blockade. Clin. Cancer Res..

[46] Bertino JR, Waud WR, Parker WB, Lubin M (2011). Targeting tumors that lack methylthioadenosine phosphorylase (MTAP) activity: current strategies. Cancer Biol. Ther..

[47] Tang B, Testa JR, Kruger WD (2012). Increasing the therapeutic index of 5-fluorouracil and 6-thioguanine by targeting loss of MTAP in tumor cells. Cancer Biol. Ther..

[48] Tang, B., Lee, H. O., An, S. S., Cai, K. Q. & Kruger, W. D. Specific targeting of MTAP-deleted tumors with a combination of 2’-fluoroadenine and 5’-methylthioadenosine. *Cancer Res*. 10.1158/0008-5472.CAN-18-0814 (2018). 10.1158/0008-5472.CAN-18-0814 PMC6072572 29844120

[49] Chen CH (2014). A peptide that inhibits function of myristoylated alanine-rich C kinase substrate (MARCKS) reduces lung cancer metastasis. Oncogene.

[50] Chen CH (2014). Targeting myristoylated alanine-rich C kinase substrate phosphorylation site domain in lung cancer. Mech. Ther. Implic. Am. J. Respir. Crit. Care Med.

