# Supplementary material for: Targeting the insulin-like growth factor-1 receptor in MTAP-deficient renal cell carcinoma
Source: Signal Transduct Target Ther. 2019 Jan 25;4:2. doi: 10.1038/s41392-019-0035-z (PMC6345872; doi:10.1038/s41392-019-0035-z)
Supplement: Supplementary file 1 — Supplemental Material_clean [file 41392_2019_35_MOESM1_ESM.docx]

**Online Data Supplement** for

Targeting the insulin-like growth factor-1 receptor in MTAP-deficient renal cell carcinoma

Jihao Xu^#^, Wen-Hsin Chang^#^, Lon Wolf R. Fong, Robert H. Weiss, Sung-Liang Yu, Ching-Hsien Chen^*^

**Supplementary Methods**

**Reagents and antibodies**

Dulbecco's Modified Eagle's medium, RPMI-1640 medium, fetal bovine serum and penicillin-streptomycin were purchased from Life Technologies Inc. (Carlsbad, CA). Lipofect-AMINE™ was purchased from Invitrogen (Carlsbad, CA). VECTASTAIN® Elite ABC Kit (Rabbit IgG), VECTOR® Hematoxylin QS nuclear counterstain and DAB solution were purchased from VECTOR Laboratories Inc. (Burlingame, CA). Linsitinib (OSI-906) was purchased from Selleckchem (Houston, TX). Anti-dimethyl-arginine antibodies, symmetric (SYM10) and asymmetric (ASYM25), were purchased from EMD Millipore (Burlington, MA). Anti-MTAP antibody (2G4) was purchased from Novus Biologicals (Littleton, CO). Anti-phosphotyrosine (4G10) antibody was purchased from Upstate Biotechnology, Inc. (Lake Placid, NY). Anti-pTyr1131 IGF1R, anti-IGF1R, anti-pTyr705 STAT3, anti-STAT3, anti-pTyr416 Src, anti-Src, anti-pThr202/Tyr204 ERK1/2, anti-monomethyl-arginine (D5A12) and anti-β-actin antibodies were purchased from Cell Signaling Technology, Inc. (Danvers, MA).

**Plasmid constructs and primers**

The V5-tagged MTAP was expressed in mammalian cells by using pcDNA3.1/V5-His TOPO construct (Invitrogen, Carlsbad, CA). The MTAP coding region was amplified by PCR using the forward primer: 5’-GATATCATGGCCTCTGGAACAACC-3’, which introduced an EcoRV site, and the reverse primer: 5’-CTCGAGGCATGTCTTGGTAATAAAACAGAA-3’, which introduced an XhoI site. The amplified product was cloned into the constitutive mammalian expression vector pcDNA3.1/V5-His TOPO. The cDNA was then fully sequenced to ensure that no mutations were introduced during the PCR amplification. The MTAP D220A mutant was generated using site-directed mutagenesis (Stratagene, La Jolla, CA) by overlapping extension using PCR with mutagenic primers. The primer sequences for site direct mutagenesis, forward: 5’-GTATCGCCATGGCGACAGCCTATGACTGCTGGAAGG-3’, reverse: 5’-CCTTCCAGCAGTCATAGGCTGTCGCCATGGCGATAC-3’. The resulting mutations were confirmed by Sanger DNA sequencing. For generation of plasmid for MTAP knockout, the insert oligonucleotides, oligo 1: 5’-CACCGTCATCTCACCTTCACGGCGG-3’ and oligo 2: 5’-AAACCCGCCGTGAAGGTGAGATGAC-3’, were synthesized, annealed and cloned into the LentiCRISPRv2 expression vector according to the manufacturer’s protocol.

## Cell culture and transfection

The kidney cancer cell lines, Caki-1 (VHL-positive), ACHN (VHL-positive), TK-10 (VHL-positive), 786-O (VHL-negative) and A498 (VHL-negative) cells were purchased from the American Type Culture Collection (ATCC) (Manassas, VA), which performs cell line characterizations, and passaged in our laboratory for fewer than 6 months after receipt. Normal human primary kidney tubular epithelial (NHK) cells were purchased from Lonza (Allendale, NJ). Both normal and cancer cell lines were cultured in Dulbecco's Modified Eagle's medium with 10% fetal bovine serum and 1% penicillin-streptomycin at 37°C in a humidified atmosphere of 5% CO_2_. For ectopic expression of V5-tagged MTAP in MTAP-deleted cells, ACHN cells were transfected with pcDNA3.1/V5-His TOPO-MTAP wild type, pcDNA3.1/V5-His TOPO-MTAP D220A mutant, or pcDNA3.1/V5-His TOPO vector using Lipofectamine 2000 reagent (Invitrogen, Carlsbad, CA), according to the manufacturer’s protocol. For establishment of MTAP-knockout stable cell lines, we utilized lentiviruses that were generated by co-transfection of HEK293T cells with the appropriate MTAP sgRNA-containing lentiviral vector and a packing DNA mix, using Lipofectamine 2000. Cells were infected at three different Multiplicities of Infection (MOIs) in polybrene (8 µg/mL)-containing medium. Twenty-four hours after infection, the cells were treated with puromycin (final concentration 2 µg/mL) and puromycin-resistant clones were selected and sequenced to confirm the gene-editing results. For siRNAs transfection, ON-TARGETplus MTAP siRNA and scrambled siRNA sequences (Thermo Scientific) were transfected using DharmaFECT according to the manufacturer’s protocol.

## Patient tumor specimens and immunohistochemical staining

Kidney tumors (which include 56 patients) were obtained from patients with histologically confirmed RCC who underwent surgical resection at the UC Davis Comprehensive Cancer Center (Sacramento, CA). This investigation was approved by the Institutional Review Board of the UC Davis Health System. Written informed consent was obtained from all patients. Formalin-fixed and paraffin-embedded specimens were used, and immunohistochemical staining was performed for MTAP expression. Detailed experimental procedures were modified from the paraffin immunohistochemistry protocol supplied by the manufacturer (Cell Signaling, Danvers, MA). The slides were de-paraffinized in xylene and rehydrated in graded alcohol and water. An antigen retrieval step (10 nM sodium citrate (pH 6.0) at a sub-boiling temperature) was used for each primary antibody. Endogenous peroxidase activity was blocked by 3% hydrogen peroxide followed by blocking serum and incubation with appropriate antibodies overnight at 4°C. Detection of immunostaining was carried out by using the VECTASTAIN^®^ ABC system, according to the manufacturer’s instructions (Vector Laboratories, Burlingame, CA). A four-point staining intensity scoring system was devised to confirm the relative expression of MTAP in cancer specimens; scores ranged from zero (no expression) to 3 (highest-intensity staining) as described previously[^1-4^](#_ENREF_1). The results were classified into two groups according to the intensity and extent of staining: in the low-expression group, staining was observed in 0–1% of the cells (staining intensity score = 0), in less than 10% of the cells (staining intensity score =1), or in 10%-25% of the cells (staining intensity score = 2); in the high-expression group, staining was present more than 25% of the cells (staining intensity score = 3).

**Supplementary Data**

**Table S1.** Summary of clinicopathologic features according to MTAP expression.

| **Characteristic** | **High**  No. of Patients (%) | **Low**  No. of Patients (%) | ***p-*value** |
| --- | --- | --- | --- |
| **Number of patients** | n=25 | n=31 |  |
| **Age (mean$\pm$SD)** | 58 $\pm$ 13 | 61 $\pm$ 13 | 0.37^†^ |
| **Gender** |  |  |  |
| Male | 15 (27) | 18 (32) | 0.89^‡^ |
| Female | 10 (18) | 13 (23) |  |
| **Race** |  |  | 0.005^‡^ |
| Caucasian | 12 (21) | 17 (30) |  |
| Mexican American  African American  Other | 8 (14)  2 (4)  3 (6) | 1 (2)  1 (2)  12 (21) |  |
| **Grade*** |  |  | < 0.0001^‡^ |
| G1 | 12 (24) | 4 (8) |  |
| G2  G3  G4 | 10 (20)  0 (0)  0 (0) | 9 (18)  14 (28)  1 (2) |  |
|  |  |  |  |
| ^†^T test.  ^‡^Fisher’s exact test. | | | |

*Some patients without grade information

**
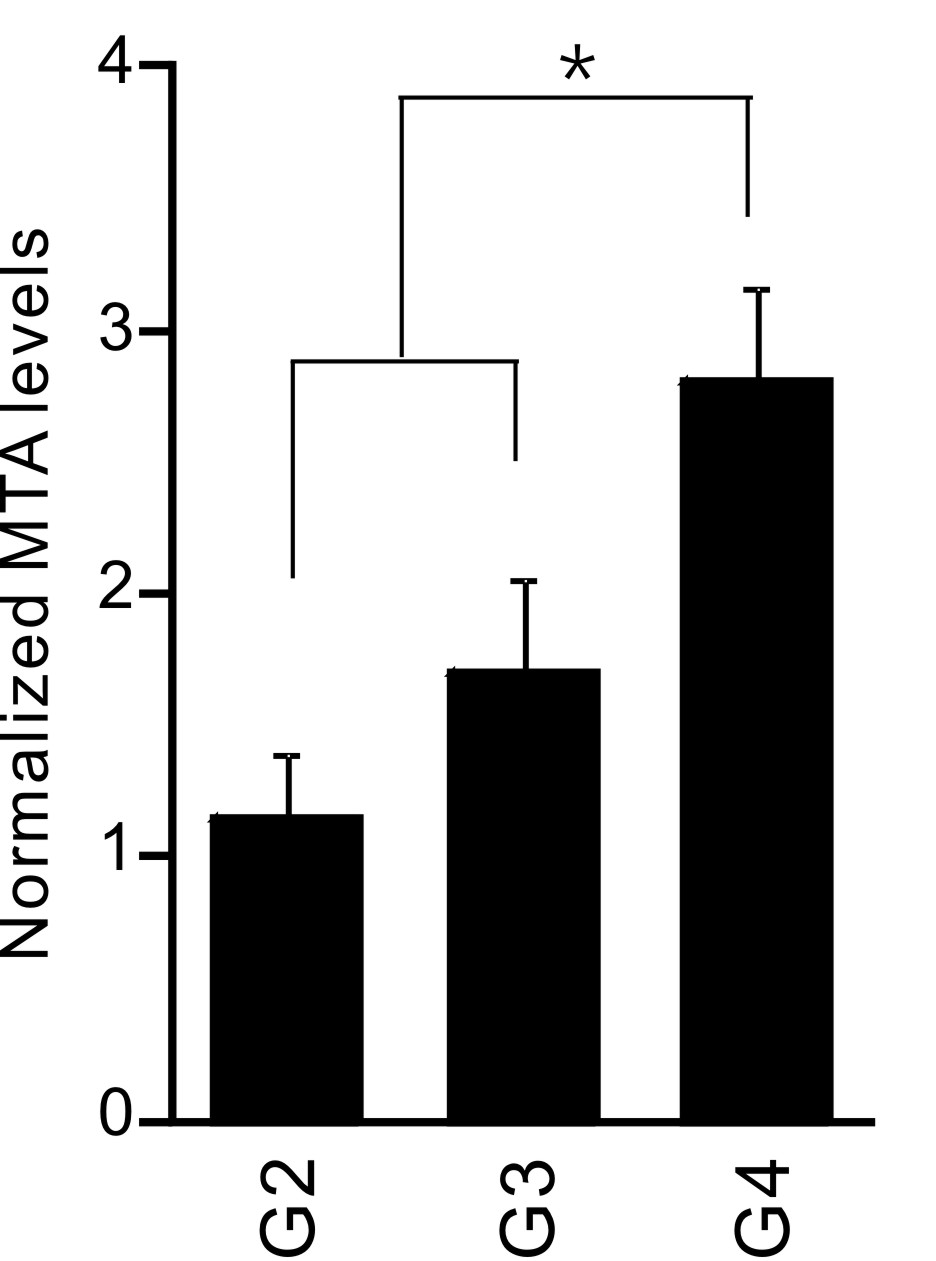
**

**Figure S1. Analysis of metabolite levels in RCC samples from the TCGA dataset.** S-methyl-5-thioadenosine (MTA) levels in RCC tissues with grades 2 (G2), 3 (G3) and 4 (G4) are analyzed. *, *p* < 0.05.

**
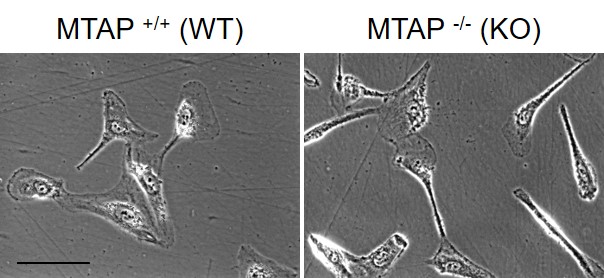
**

**Figure S2.** Effect of MTAP expression on cell morphology in 786-O cells. After 24 hours of cell seeding, MTAP-knockout (KO) and wild type (WT) 786-O cells were examined by a phase contrast microscope. Scale bars, 10 μm.

**
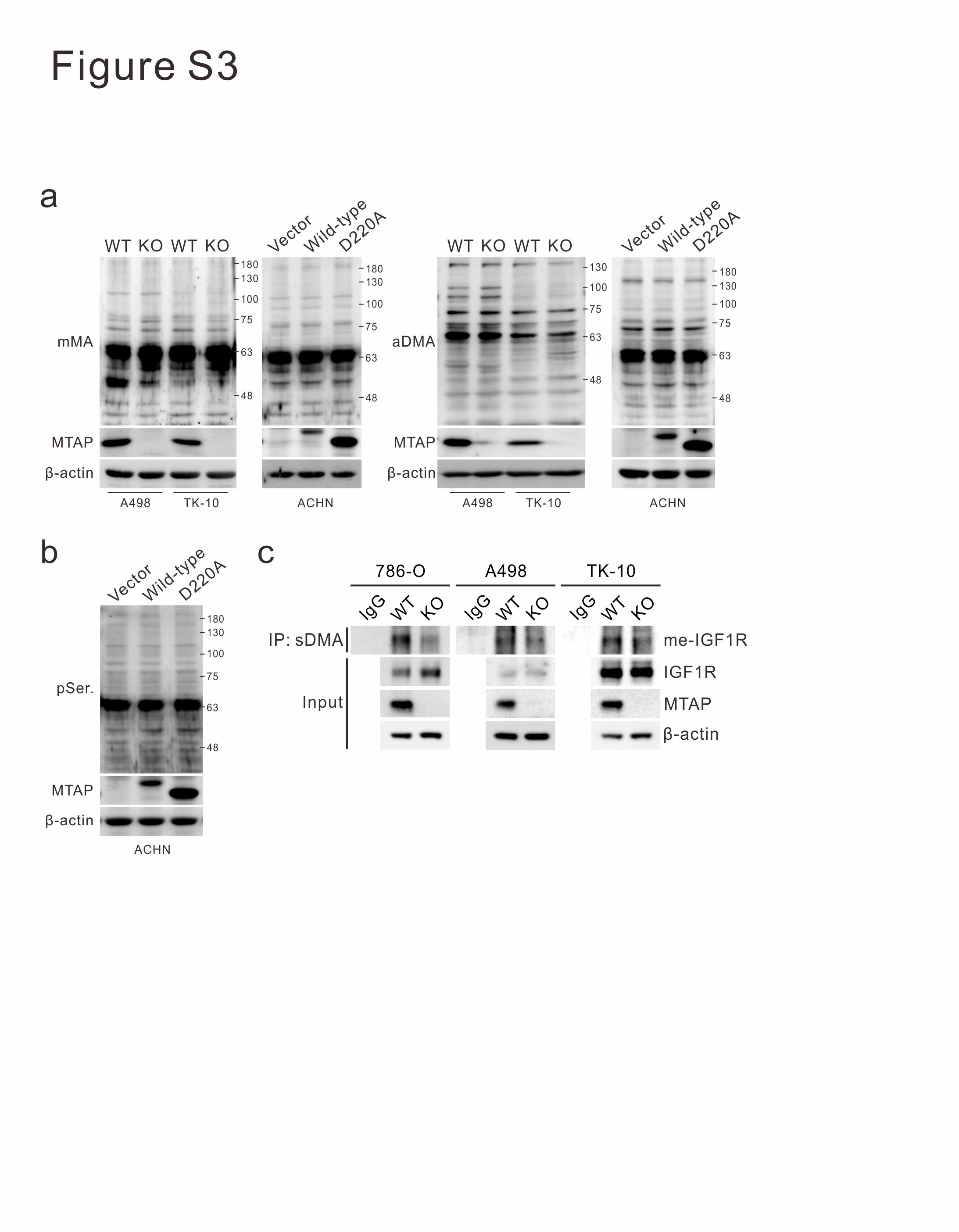
**

**Figure S3.** Cell lysates from MTAP-knockout or -overexpressed RCC cell lines (786-O, A498, TK-10, ACHN) were subjected to Western blots for measurement of the levels of mMA, aDMA (**a**) and phospho-serine (pSer.) (**b**). Determination of protein arginine methylation levels in various RCC cells by using immunoprecipitation and immunoblotting with indicated antibodies (**c**).

**Supplementary References**

1. Kuo TC, Tan CT, Chang YW *et al.* Angiopoietin-like protein 1 suppresses SLUG to inhibit cancer cell motility. *J Clin Invest* 2013; **123**:1082-1095.

2. Chen CH, Chiu CL, Adler KB, Wu R. A novel predictor of cancer malignancy: up-regulation of myristoylated alanine-rich C kinase substrate phosphorylation in lung cancer. *American journal of respiratory and critical care medicine* 2014; **189**:1002-1004.

3. Chen CH, Statt S, Chiu CL *et al.* Targeting myristoylated alanine-rich C kinase substrate phosphorylation site domain in lung cancer. Mechanisms and therapeutic implications. *American journal of respiratory and critical care medicine* 2014; **190**:1127-1138.

4. Chen CH, Cheng CT, Yuan Y *et al.* Elevated MARCKS phosphorylation contributes to unresponsiveness of breast cancer to paclitaxel treatment. *Oncotarget* 2015; **6**:15194-15208.
